# The Physician in the Making1Lecture delivered at the opening exercises of the 59th session of the Medical Department of the University of Buffalo, September 26, 1904.

**Published:** 1904-11

**Authors:** T. H. McKee

**Affiliations:** Buffalo, N. Y.


### BUFFALO MEDICAL JOURNAL.

VOL. XLIV.—LX. NOVEMBER, 1904. No. 4

### ORIGINAL COMMUNICATIONS.

### The Physician in the Making.[1]

T. H. McKEE, M. D., Buffalo, N. Y.

*Mr. Chairman, Gentlemen of the Faculty, Ladies and Gentlemen:*

I FEEL I may claim your indulgence in permitting me to say that in being asked to deliver the opening lecture of this term, and to fulfil a function which so many of my seniors could have graced with their oratory and improved by their wisdom, I appreciate the consideration, shown not to the individual, but to those younger members of the teaching staff whom it is my privilege to number among my personal friends and daily associates; and who, as everyone knows, are not becoming wealthy in a purely material sense as a result of their scholastic labors, but who, nevertheless, are "laying up treasure where thieves will not break in to steal, nor moth nor rust corrupt"—treasures not alone of an intellectual character, but gems of hearty good-will and real friendship, so freely accorded us by the members of our classes, and whose memories, we know, when the autumn days shall come, will far outweigh any recollection of the temporary inconveniences we are sometimes called upon to incur.

Under those circumstances, I have departed somewhat from the usual lines in the selection of a subject, and instead of a more pretentious effort, have elected to recapitulate in brief some of the topics relating to the Physician in the Making, which we so often discuss informally among ourselves, asking you to remember that progress is ever conditioned on discontent. I must also state in fairness, that I can claim no originality for the views expressed, nor do I know they are anybody's else than my own; though with some of them I believe many of my colleagues are

1. Lecture delivered at the opening exercises of the 59th session of the Medical Department of the University of Buffalo, September 26, 1904.

in accord; believing also in the principle which John Bright so well crystalised in the statement that two men could not discuss a public issue without creating political power, it seems as though the ideas of earnest men with regard to matters of their daily work might prove worthy of a hearing, even on an occasion so dignified as the opening of the fifty-ninth session of a school known throughout the length and breadth of the United States, not for its heavy endowments, or costly appurtenances, but is solely and justly celebrated for the good work it has done, and the distinguished names that have been associated with it.

The first essential which every business man would take into consideration in preparing for the manufacture of any finished product would be the selection of the material. Up to the very recent past, little or no care was exercised in the selection of the material for the manufacture of a product on which were to rest responsibilities which would break the average statesman, and of which would be demanded a fund of charity, of tenderness and fidelity to altruistic principles that would bankrupt the priesthood. The only prerequisite demanded of the candidate for the honor of carrying on a daily warfare with disease and death, of being made the recipient of the pain-wrung secrets of the heart's most holy of holies, of being made the arbiter of the social standing of half the community, was sufficient funds to buy his tickets, or failing that, the ability to furnish approved joint notes for the same. In most states of the Union that has been changed in a very large measure, though to our national discredit be it said, not in all; and among those which demand the highest standards for matriculation into the study of medicine, is to be found the state of New York. Notwithstanding the advances made, much remains to be done, and in order that it be done well and quickly, a healthy public opinion must be aroused, not alone in the profession, and among educational bodies generally, but in that great court of last resort in every democracy, among the masses of the laymen.

Owing to the extraordinary overflow of medical science in all directions, to advances in technic, in the knowledge and use of instruments of precision, but particularly in the ever increasing range of knowledge demanded of collateral branches, especially physics, mathematics and biology, it has become quite impossible to work up much of the material that was once offered,—and not more than ten or fifteen years ago either,—into an article that any self-respecting firm would care to send out under the ægis of its trade mark. The man who cannot place his hand to paper without evidencing his ignorance of the mother tongue is no longer regarded as a desirable recruit to the ranks of the profession. Those of us who have been teaching long enough can bear witness to the marked betterment of the material offered with every increase of matriculation requirements. And that is the logical place for the process of weeding to be carried out. It is a distinct hardship and an injustice to admit a young man to a costly course of study only to be cast out later because the teaching staff and the faculty cannot supply those essentials which alone can insure his success, and which have been denied him by nature, or a defective primary education. But it is an infinitely greater injustice, and one involving a grave question of complicity in murder before the fact, to turn such an individual loose upon the community after a long and wholly unsatisfactory course of training; for the university and state examinations cannot be relied upon to eliminate the incompetent. A clever coach can circumvent any permanent examining board whose questions he has facilities for studying from year to year. If he escape the matriculation examinations, then he should not escape his first and second university tests.

In the good old days mentioned, it was possible for a man to learn nearly all the useful, practical knowledge of the science and art of healing that was commonly known during his college course; now the man does not live who could keep abreast of the daily advances in all the departments of medicine, even if he were not compelled to spend any time in its practice. The best, therefore, that a modern medical school can hope to do, is to lay a foundation which shall be broad and deep, and on which the man who is worthy to be admitted to our ranks shall strive to erect a superstructure as long as life shall last, laboring always in the full consciousness that he can never, never, hope to see it finished. In order merely to lay that foundation, it is necessary to ask that certain subjects now taught in the colleges shall be relegated to the primary schools where they belong, that the time may be better utilised in teaching the purely technical branches. One weak spot in the selection of our material lies in the fact that the student is allowed to make up the counts necessary for matriculation by taking examinations in a variety of subjects having little bearing on his subsequent professional work, to the neglect of others essentially necessary to his medical student career.

Very few young men go through the high schools nowadays without having fully determined upon their future line of work; it would therefore be no hardship, but a positive benefit to them, if that course were so arranged, having due regard to the necessities of a liberal education, as to have a direct bearing upon law or medicine; for instance, in the latter case, making a satisfactory standing in chemistry, physics and histology, a *sine qua non* to graduation from the one, and matriculation to the other. It is a question if elementary histology should not be made a part of the high school course in order that it may be a part of the mental furniture of every layman claiming so much of a liberal education. If that were done, we should have much easier work in dealing with questions of public health and sanitation, and a fair estimate of the physician's efforts along those and other lines. It is folly to expect a man to understand the rationale of the application of certain advances in modern therapeutics unless he is well grounded in physics; it is worse folly to attempt to teach either physics or elementary chemistry in a medical school, where we have more purely medical and physiological chemistry to handle than we have time to deal with. A very little intelligent coöperation between medical and lay educators should be sufficient to evolve a course which would result in bringing young men into the professional schools exceptionally well qualified to take up their special work, and save a very large part of the time now wasted during the first two years of their student lives.

Having selected your material, the next problem is to determine upon the best and most economical means of working it up into a finished product of the highest possible excellence. Heretofore medical schools have endeavored to keep pace with the spread of scientific knowledge by two very simple methods; one, lengthening the course; the other, piling more and more work upon the unhappy wretches under their jurisdiction. But the time has come when we can see that there must be an end to both processes. There is an age limit beyond which we shall not be able to go in withholding degrees; moreover, and of far more serious import, there is a physiologic limit beyond which you cannot push a jaded organism. On picking up a schedule for a certain medical college, located within a thousand miles of New York city, and, judging from the distinguished names on its governing board, one of the best administered in the country, and opening it at random, we find that during the semester for which it was issued, for three consecutive days a week, a certain class was required to be in attendance from 8.30 in the morning until 6 o'clock at night, with an interim of ninety minutes for lunch. On the fourth consecutive day, they put in the same amount of time, less one hour. When you stop to consider the size of the classes, the badly ventilated rooms in winter time, the close and concentrated attention which they are called upon to give their work, with other discomforts too numerous to mention, that the remainder of their time must be spent in equally unwholesome hospital wards, that schedule looks very like an attempt at manslaughter. The man or woman does not live who can be bombarded with facts *a la Gradgrind,* so many hours a day, week after week, and month after month, and retain or assimilate one quarter of them. It is a sinful waste of good gray matter, as evidenced by the number of cases bordering on, or quite over the line of, neurasthenia which come under our care near the close of every term.

If the essentials of medical education are ever widening, while human limitations are not correspondingly elastic, the whole problem resolves itself into a very simple question of lightering ship,—something must go overboard,—elementary chemistry first; then, I think, it would be well to send the greater part of materia medica with it. I appreciate the thrill of horror that at this moment is coursing along the central nervous systems of certain of my good friends whose hearts are wedded to this subject, but under present existing conditions, where the essential principles of drugs are universally bought, prepared with a skill and accuracy by machinery, that we can never hope to imitate by hand, there is no more excuse for making a student spend valuable time learning to roll pills, because he will use pills in his business, than in making him study the process of rolling steel rods because he will use knives in his business. Personally, I cheerfully confess to little knowledge, and less love, for that time-honored subject; and were it not for the risk of causing some embarrassment, I should like to ask some of the gentlemen present, who are enjoying so well deserved distinction in their several lines of work, how much of the old lectures on materia medica they could now recall; how often they have been called upon during their professional career to brew a pot of herbs or rub up an emulsion; and, if they were called upon, if they thought they could do it. Moreover, and I realise that this will be considered still more anarchistic and heretical, I believe that the course in therapeutics could be lightened. We are endeavoring to teach too many drugs purely as a matter of memory. I should like to ask some of the gentlemen present, who have very large practices, how many of them use twenty-five different drugs in their daily work. And yet we try to grind in a hundred during a single term. The drug idea is being too insistently impressed upon the student; in many cases to the exclusion of the various non-chemical remedial measures of equal or greater efficacy, which come to occupy an almost wholly neglected niche in his mental museum by the time he graduates. On being presented at the bedside of a sick baby, his first thought is: "What drugs can I use?" Then he begins to feel the erectores pilorum beginning to contract along his spine as he wonders how he is going to apply Young's rule for dosage to a child a very small fraction of a year old; whereas, his first consideration should be at every bedside, how far can he go without using drugs. None of the dangers which beset the process of the physician in the making is more worthy to be sedulously guarded against than that of falling a victim to the drug habit.

No part of the process of manufacture is more important than the methods employed and the character of the workmanship. A very fair article can be produced by a skilled craftsman out of indifferent material; but poor methods and faulty workmanship will ruin the very best material and render the finished product correspondingly unfit. There is no profession, not excepting even the science and art of healing, that has made more substantial progress during the last generation than that of teaching. Though a comparatively young man, I can distinctly remember when training schools for teachers were discussed with contemptuous derision; when it was considered that anyone who could pass an examination in a certain amount of book-learning, including the three R's, of course, was a fit person to be intrusted with the education of a child, no matter whether that individual had a single elementary idea as to how that child was to be taught; for instance, the relationship of numbers, or to associate the written sign with the spoken word; having no ideas with regard to discipline except one hard and fast rule that "licking" was a proper corrective for any and all dispositions and offences, with never a thought as to the moral side of the child's nature, or of the awful responsibilities that devolve upon anyone who is entrusted for a single hour with the care of a developing human mind. Whether the pupil acquired accuracy of thought and consistency of purpose was a matter of chance or heredity; kindergartens and manual training were unknown. This has all been changed in a large measure, so that now there are few communities where teaching experience is not demanded and fewer commonwealths which do not make some effort to supply that qualification, together with methods of applying their art, before turning teachers out to mangle the tender faculties of youth. But we have yet to hear of the establishment in any country of a training school for medical college teachers; yet, considering the disadvantages of environment under which they are compelled to work, and the far reaching consequences depending upon the quality of that work, where is a higher degree of skill to be demanded? It is now only in medical schools that this barbaric custom obtains, though it must be said, in justice, that much progress has been made along these lines in some of them. Still, partly due to inertia, partly to ignorance of the necessity, and perhaps more to the interposition of difficulties which, under hitherto existing conditions, were considered well nigh insuperable, they still lag far behind in teaching methods. In them we find that fallacy still nourished and practised, that because a man knows a great deal about a subject, he should be considered capable of teaching that subject; whereas, it is a well recognised principle that a plethora of knowledge, particularly in one who is far removed in his mental plane from the untrained mind of the student, may absolutely unfit him for teaching anything.

The exaggerated, timeworn, didactic method, so fittingly caricatured by Mark Twain in the person of the old German professor who at the sound of a gong ran up on the rostrum and lectured away for an hour as hard as he could to an audience of one or two students with apparently never a thought as to their mental attitude, and at the sound of the gong ran down again, is giving way slowly but surely to living, breathing, heart-to-heart methods, where the mind of the student comes into close and sympathetic contact with that of the teacher; where difficulties are freely discussed between them to the immense benefit of the benches and not a little to the educative improvement of the chair. But it seems to me we have not gone nearly far enough yet in retracing our steps toward kindergarten methods. I am using that word advisedly and literally, believing that the mind of the medical student entering upon the exploration of wholly new and untried fields should be regarded as the mind of a child of larger growth, and be treated accordingly. For instance, one of the most irrational features among the many inconsistencies that beset our educational system is the idea that the beginner, with the wholly uncultivated mind, whose uncertain footsteps lead him deeper and deeper into intellectual jungles, is to be delivered over to the tender mercies of the least experienced, and least skilful member of the teaching staff; whereas, common sense demands that he who is least able to help himself should be under the care of him who is best able to help him. Now, I do not mean to advocate that our honored Dean and Dr. Stockton shall be assigned to the cultivation of freshmen intellects this term, leaving the seniors to us; for, however highly complimentary that would be to those gentlemen, there are other things which have to be taken into consideration,—but that is another story. However, I challenge criticism on the principle involved.

Unquestionably, a very large part of the freshmen's year, and to a less degree of the sophomores, is irrevocably lost to him in the effort to find his feet, to learn what he is to do and how he is to do it. Huxley has summed up his difficulties most admirably in the statement, "What the student wants is a professor who shall stand between him and the infinite variety and diversity of human knowledge." If you will substitute the word "medical"

for "human," you have pointed out to you practically the whole duty of the medical college teacher. Like the nursling, it is the quality not quantity of his mental food which will determine the character and extent of his intellectual growth; the judge of that quality cannot be the untried mind of the beginner, but the ripest effort of the man who has probably earned his capability in the dearest schools of experience. He alone can inculcate the habit of reasoning from given data all the information that can be acquired by that method. Our young men come up to the universities filled with book knowledge, but with too little live, practical acquaintance with either natural or scientific phenomena.

The student is filled with a slavish adoration for authorities, and unless that be jarred out of him during his college days, he will carry it to the solution of his medical problems, by looking up the opinions of some celebrated individual instead of examining each case *au fond,* and on its own merits; and when at last he graduates, and comes face to face with bedside conditions, which never will play fair and according to rule, he stands helpless and appalled before the sacrilegious liberties which pathology presumes to take with the teachings of his distinguished preceptors.

In these days of pandemic itch for writing of voluminous literature on all subjects, seemingly from every possible divergent point of view, not the least important function of university training, whether in arts or medicine, but particularly in the latter, is to teach the student to assay opinions with all the skill of an expert analyst, that he may readily and accurately separate the dross from the pure gold. It may be objected that the education of these faculties does not belong to the medical, but to the primary school. That is true, and in proportion to the thoroughness with which it has been done, will you be relieved from that duty; and there again is one of the very best reasons for still higher matriculation requirements. But we are here to face conditions, not theories. I feel assured that every member of the teaching staff will bear me out in the assertion that during the first two years our students manifest an extraordinary and lamentable lack of training in accuracy of thought and expression. And that defect is not confined to this state or country. Apropos to this point, Professor Gilman quotes the opinions of a well-known English medical educator, as follows: "A distinguished anatomist whose daily duty it is to train educated classes of aspiring physicians—everyone a college graduate—in the knowledge of the human body, told me that his hardest task was to make the members of his classes see three dimensions. These medical students, who must learn the topography of every human organ, are so used to the printed page, to diagrams and other flat illustrations, that they cannot perceive solidity nor comprehend structure until their bad habits are broken up, their power of vision and image-building recovered."

It is in exactly this connection that some half dozen of the foremost colleges in the country have made a long, long step forward of extreme educative value by a resumption of kindergarten methods,—that is by the introduction of clay-modeling in the medical schools. It needs but a moment's thought to show the wide application of this simple process to the study of anatomy. You need but recall your student days to regret the hours and hours of practically wasted effort in trying to glean and to store a few concepts of gross cerebral anatomy from illustrations in one dimension, which always seemed to depict the things which you did not want to see, and left undepicted the things which you did want to see; and when you turned to the printed page, confusion was worse confounded. In one quarter of the time which you wasted in an effort to get ideas, more or less inaccurate, and which never would stay with you, you could have had accurate knowledge impressed upon you in the most vivid way in the world over a child's modeling board.

And so we find evidence of progress of the most hopeful character all along the line, and considering the fact that the process of making the physician must necessarily be continuous, one of the most encouraging features of this educational renaissance, is the progressive spirit manifested of late by contributors of periodical medical literature. Perhaps no other agent has a greater post-graduate effect on the professional character of the physician than his medical journal, if he respect its opinions.

I regret that I am not in a position to speak clearly and comprehensively with regard to the proposed innovation on the part of our old and honored friend, the BUFFALO MEDICAL JOURNAL, a change which cannot help increasing, not only its interest, but its educational efficiency in very large measure. It deserves the warmest sympathy and support of all who have the elevation and security of the profession at heart aside from all purely personal desire for intellectual improvement.

Another defect in our present process of manufacturing, and one which from the nature of his surroundings is often unnoticed in college course, but which may lead to no little embarrassment after he goes out into the world, is the saturation of his mind with abnormal conditions. He gets so accustomed to them that it never occurs to him that anything brought to his attention can be normal; in fact, he is too often wholly ignorant of the appearance of many common, normal, vital phenomena. He is instructed with regard to all sorts of abnormal pulse conditions, but may not recognise a normal one when he feels it, especially under varying circumstances.

Like the arts colleges, medical schools are open to the criticism that they turn out graduates trained with too little regard for the practical requirements of life. From start to finish the student finds his curriculum loaded with technical matter, but with hardly a word of instruction for the commonplaces of everyday professional life, in the acquirement of which by haphazard, he will lose golden opportunities, while the college and profession lose more valuable opportunities to ground him in certain simple principles of conduct, which may mean all the difference between the successful man earning an honest living, and a disappointed, unsuccessful practitioner who sinks to the level of the advertising fakir to practise upon the infirmities of the ignorant. To many of us it seems highly inexpedient and unbusinesslike to accept a man for a long and expensive course of training for a profession to which he is expected to sacrifice the remainder of his life, to which he must look not only for his daily bread, but for provision for those who are near and dear to him after the working tools shall have fallen from his own grasp, and to send that man out without a single, definite idea as to the value of the wares he has to sell. The average college graduate, unless he chance to have been under the supervision of a preceptor, which is now very rarely the case, does not know when he opens his office whether he is entitled to fifty cents, a dollar, or three dollars for a given piece of work. His soul is torn with doubt whether he shall attempt to establish a fifty cent or a dollar rate for office calls; and if he has had no business experience, which is more than likely, and little knowledge of human nature, and chance to settle in a neighborhood where experienced men are prostituting their skill for fifty cents a visit, he will sink to that level, all unconscious of the fact that he is losing a brilliant opportunity to advertise himself by charging a little more than his neighbors, provided he has a good article to sell, and still keep within reasonable limits. In the first place, it will get him noticed, and in the second place, if he does good work, the better element of the community will respect him more for demanding a reasonable return for his long and expensive course of training.

He has never been taught that he should charge more where he is compelled to do a surgical dressing than for an ordinary visit; that in prescribing drugs "in the original package" he is filling the pockets of the proprietary medicine man while cutting the ground from under his own feet; he does not know that when he pulls a patient out of the grip of death by poison he should charge for a major operation, which he has really performed, and not three dollars for a night call; he does not know that it is his duty earnestly to strive by precept and example to educate the unthinking public to the appreciation that it requires a higher degree of skill and a finer quality of judgment to carry a patient through a long period of critical expectancy terminating in a successful accouchement than to amputate an arm or a leg, and that he should be paid accordingly.

It may be objected that these matters are not proper subjects for a medical course. That is altogether too narrow a view. Everything that he does not know and must necessarily use in his daily practice is a proper subject for either direct or indirect instruction, especially where the welfare and respect of the profession at large may be injured by his innocent ignorance. What right have you, as old practitioners, to expect a young man to abide by rules of ethics of which he has never heard? How can you demand that he shall conduct himself with correctness, for instance, in the matter of a consultation involving questions of conduct with which he is wholly unfamiliar, many of the problems confronting him being of so delicate a character as to test the diplomacy and ethical courage of which the best of you are capable?

A very large proportion of the evils which the profession is heir to can be eliminated, or rather avoided, by proper care of the physician in the making. Owing to the constantly increasing demands of matriculation requirements we are getting a far better quality of students than those who entered ten or fifteen years ago; today they are largely high school boys, or young college graduates, whose mental and moral habits are far from being firmly fixed. They are still more susceptible to molding influences than the older men who a generation ago, having saved sufficient money to invest in a perpetual ticket, forsook the plow or the district school to take up a medical career destined in some cases, it must not be forgotten, to add new stars of the first magnitude to the professional firmament, but too often in others, providing additions to the ranks of the advertisers, or others who, while not criminally inclined, were still incapable of adding to the standard of the profession in the minds of those with whom they were to be associated. The advertising shark, who filches his illgotten gains from the sick and suffering under pretence of curing incurable diseases, the criminal practitioner, and the man who knows nothing and cares nothing for the rules of honorable medical intercourse can be treated effectually in one way,—and in one way only,—and that is by cutting off the supply. Statute law has again and again proved its inefficiency in this respect; the work must be done in the colleges. It may be objected that in the present overloaded state of our schedules, time cannot be found for an extended course of lectures on medical ethics. We would reply that no part of the course is more worthy of serious consideration; yet if time and opportunity afforded, that would be the least effective and most unsatisfactory way of teaching these truths. Every member of the teaching staff should be made to feel it his duty on the thousand and one occasions which will arise during the student's four years' attendance to inculcate those principles of fair dealing, that recognition of his responsibility, first to his patient, then to the state, to his alma mater, and, lastly, to the *ego* with whom he is obliged to retire in the silent watches of the night, and which if well learned, or better, woven into the web and woof of his professional fabric, shall be all sufficient to keep his feet in the straight and narrow way beaten by his illustrious predecessors who have lifted his profession to its present high estate. It would be undesirable in the extreme to have anyone burdened with the teaching of these matters; everybody should teach them in self-preservation.

While it is easy to criticise, easy to suggest improvements in educational work, it is not so easy to explain how it shall be done. Among all the measures that have proven to be most efficient in developing a higher grade of teaching service, of developing and keeping up that *esprit de corps* that has lead to so good practical results of a reformative character, and most far-reaching import, none has worked to better purpose than the frequent meetings of teachers' associations for the discussing of problems relating to their business. Even dancing masters, music teachers, and all sorts of "professors," find it advantageous to belong to local and state organisations, in order to provide for interchange of ideas, and above all, to ensure consistent and concentrated effort toward improvement in their several lines of work. All recognise the value of coöperation, apparently, except medical college teachers, who have more difficult problems to face, with less effective means of meeting them, and less preparation for their work, than any other class of educators. There is no room for doubt that incalculable good could be accomplished by having monthly meetings at least, of the teaching staff of every college for the elucidation of difficulties, the betterment of methods, and to ensure a closer correspondence among their several lines of work. How much wider would be that field of usefulness if there were state and national associations; what a tremendous leverage it would give for uplifting the cause of medical education by cementing a closer union of purpose among the different institutions of the country. It is true, efforts are being made, and which must be crowned with a considerable degree of success, to secure coöperation in the general policies of medical colleges by the organisation of such bodies as the Association of American Medical Colleges. But they cover quite another field. Let your general policy of medical education be never so harmoniously developed, no matter how well and scientifically your curricula be arranged, no matter how many hundreds of thousands you number in your endowments, the work is bound to be more or less unsatisfactory to the student, to the college, not to say tragical to the state, unless efficient teaching be done in the class rooms; and to that end you need the hearty and intelligent coöperation of all who are engaged in class-room work. Of course, there are difficulties in the way of such an accomplishment; but to those who are in real earnest, difficulties are but useful incentives to higher achievements.

If the demands which are to be made upon an article may be assumed fairly as a criterion for the process of manufacture, then more exacting attention should be given to the moral side of the physician in the making, than to his ability to pass examinations. Moreover, the people who have delegated to medical examining boards, by virtue of various statutory enactments, the right and the duty to determine the character of the individual who is to be turned loose upon them, and establish sacred relationships with them, have the right to demand that a real and effective supervision shall be exercised in that direction instead of a merely perfunctory pretence.

For some reason or other, originally more or less well founded undoubtedly, the world at large has come to the conclusion, and has maintained the opinion through many generations that medical students are what the man in the street would term "a tough lot;" and that divinity students are good little boys. As a natural result, both classes have done their best to live up to their reputation,—and with a considerable degree of success. Whether or not the average man, or woman, who is attracted to the study of medicine, in spite of the many unpleasant features incident to the work, is endowed by nature with a harder heart or less sensitive organism than his fellows, is an open question; but if it be assumed to be true, for the sake of argument, what higher tribute could be asked to the nobility of the calling, to its humanising influences than the character that the practice of that profession has developed out of confessedly crude material, with little or no encouragement in the way of training, in the long roll of illustrious men of every nation, who have been loved and admired by their contemporaries, less for their skill and brilliant intellectual achievements than for their broad charity and ready self-abnegation to the cry of distress wherever and whenever it has sounded.

To be sure, we have our beacon lights which send their white shafts to the farthest corners of civilisation, in the names of such men as Reed and Lazear, who gave up their lives a voluntary sacrifice that others might live—unsupported and unstimulated by the plaudits of an acclaiming multitude or the thunder of victorious guns, but in the cool calculations of the laboratory. But who knows or recks of the tens of thousands, who in their daily work are as surely and steadily sacrificing their physical powers in the service of the worthy and unworthy alike, in obscure country districts, in the noisome and filthy tenements of great cities unregarded, unappreciated by the rushing mammon mad throngs, yet each in his sphere, and on whom no higher encomium can be laid than to say he is a veritable Weelum MacLure.

But whatever ennobling influences are to be found in the practice of the profession, whatever its power to develop the gentler and higher attributes of the soul, it is no excuse for neglecting the moral side of the physician in the making. There is no large commercial institution in these days where heavy responsibilities are entrusted to the clerks, as in banks and trust companies, that does not exercise a more or less strict supervision over the extramural habits of its employes. And there is no shadow of excuse for the faculty of a college that has any conception of its responsibilities to the state, knowing less of the moral character of its young men, after four years' residence, than would a financial corporation after four weeks' employment. And yet there are graduated, not infrequently, from almost every college in the country, men whom their classmates would not think of introducing to their families. The policy is bad, bad from a purely business point of view, for the world is outgrowing crudities, and is demanding better finished products along ethical as well as physical lines; and the man or institution that aims at anything more than success of the most ephemeral character would do well to keep in mind certain fundamental principles. Modern social conditions, in other words the sociological status of the world is not the result of arbitrary enactments by any race or people, but is the product of a process of ethical evolution as certain and immutable in its results as those physical developments which have determined the physiological status of the human hand or the human lung. The basis of that physical evolutionary process has been the preservation and perfection of the race; and the basis of the ethical process synchronous and in harmony with the physical was condensed in that admonition from the lips of the world's greatest teacher, a sentence more pregnant with far-reaching philosophy than any other in recorded literature: "Do unto others as ye would that they should do unto you."

I think this defect can be easily remedied by the very simple process of getting the college managements in closer touch with their students; an acquaintanceship which would be of vast benefit to the student, while the faculties would often learn things that would do them no harm to know.

As proof positive that the bad repute of the medical student is extrinsic, rather than intrinsic, is due primarily to the atmosphere that is thrown about him, you have but to notice the great change which takes place as soon as he enters his senior year. It is not due to the fact that new men are lecturing to him; he has been hearing most of them in all probability for a year or two; it is not due to difference in the character of his studies, because they are practically the same as during the past year; but his whole bearing is changed; he is more sedate; he has a dignity and air of responsibility which he never had before,—and never will again,—and it is all because he is regarded in a different light by those about him. If you demand high qualifications, you will get them, and not until you do. With improvement in the material offered, with the spread of such admirable agencies for character-building as the University Young Men's Christian Association, with the increasing influence of college fraternities, with a stricter discipline, coupled with a closer and more sympathetic acquaintance on the part of college managements, it should be possible within a few generations to live down the reputation for rascality which every young man is obliged to assume who enters a medical school.

There is yet another agency which should never be lost sight of in any educational process, and that is the subconscious influence of the teacher upon his students. Who cannot look back and single out some lives that, perhaps all unconsciously at the time, have exercised a supreme molding influence on his own? Who cannot go back to his boyhood's days, and select some teacher perhaps in an old log school-house, whom you now know,—though you did not realise it then,—exercised a powerful influence on your moral development? There was a time in the memory of some of the oldest among us when a lecturer's character appeared never to be taken into account; when if a brilliant or well-posted man he might still be given a class of students, though a drunkard or a libertine, his character a standing offense to heaven. But those conditions are happily changed, for where there was no conscience as a rule and guide, there grew up a business sense, which deemed it to be worth while for an institution, as for an individual, to preserve as fair a semblance of decency as circumstances would permit. The reason for the tremendous vitality and boundless spread of Anglo-Saxon ideas of religious liberty and representative government, of successful commercial enterprise and unlimited educational facilities, is due to the fact,—not that the little island from which they sprang was fortunately situated geographically, not that its inhabitants were a race of extraordinary mental and physical vigor,—all these advantages being found in large measure in other races who have not set their imperial stamp upon the world in all its activities,—but is due to the fact that by chance or providence, or what you like, they early got their national bark into the tideway of the eternal principles of right and progress; and ever since they have been rushing along, often against their will, it is true, toward that preëminence which they now enjoy in all that tends towards the advancement and true upbuilding of social conditions of the race. And it is so with an institution; however humble its beginning, however straightened its circumstances, if administered in accordance with the principles which conscience and common sense approve, and has vitality enough to become known of its fruits, then it is bound to go on with ever accelerating momentum towards the highest measure of success.

The whole trend of the training in such an institution will be toward making a physician fitted for leadership among men; and man secure in the accuracy of his knowledge and judgment, and who by the very exigencies of that condition must command respect and authority in his community. It is to him will ultimately be referred the disposition of its submerged members, the lunatic, the criminal, the drunkard, the pauper. His training and character will be such that he can hold his own or more with the members of other learned professions, whose callings are demanding more and more time and expense in their preparation. Our clerical friends have long had a dominating influence in educational affairs; often for their betterment, as often for their detriment. It is high time that the physician be prepared to take a commanding place in this department of public activity, thereby finding opportunities to pave the way not only for the advancement and security of his own profession, but the mental, moral, physical status of every community to which our graduates shall find their way.

91 Broadway.

---

Roosa, New York, states, (*Medical News,* Sept. 27, 1902,) euphthalmin is a valuable agent for securing transitory dilatation of the pupil, for the purposes of ophthalmoscopic examination, and harmful effects, if they ever occur, are entirely exceptional.

